# A Direct Effect of Sex Hormones on Epithelial Barrier Function in Inflammatory Bowel Disease Models

**DOI:** 10.3390/cells8030261

**Published:** 2019-03-19

**Authors:** Janine van der Giessen, C. Janneke van der Woude, Maikel P. Peppelenbosch, Gwenny M. Fuhler

**Affiliations:** Department of Gastroenterology and Hepatology, Erasmus Medical Center, 3015CE Rotterdam, The Netherlands; c.vanderwoude@erasmusmc.nl (C.J.v.d.W.); m.peppelenbosch@erasmusmc.nl (M.P.P.); g.fuhler@erasmusmc.nl (G.M.F.)

**Keywords:** inflammatory bowel disease, pregnancy, epithelial barrier, sex hormones, estrogen, progesterone

## Abstract

Background: Pregnancy is often described as an immune-tolerant state, and a disease modulatory role for pregnancy on inflammatory bowel disease (IBD) has been suggested. The direct effect of estrogen and progesterone on the intestinal epithelial barrier is underexplored. We investigated the direct consequences of these pregnancy hormones on barrier cells and their function. Methods: We used IBD patient-derived inflammatory organoid models and 2D cell lines models. Epithelial barrier function was analyzed by measuring transepithelial electrical resistance; wound closure was determined by scratch assay; and cell viability was measured by MTT assays. Pro-inflammatory cytokine production was determined by enzyme-linked immunosorbent assays. Molecular modulation of endoplasmic reticulum (ER) stress induced by tunicamycin was studied by western blot analysis of the ER stress markers GRP78, CHOP and p-IRE1. Results: Progesterone and estrogen improved wound healing and epithelial barrier function in intestinal epithelial cells via upregulation of tight junction proteins. Furthermore, these sex hormones significantly reduced ER-stress and reduce pro-inflammatory cytokine production in intestinal epithelial models. Conclusion: Our study shows that estrogen and progesterone alleviate ER stress, decrease pro-inflammatory cytokine production, stimulate wound healing, and increase barrier function of epithelial cells. Combined, these data suggest that pregnancy hormones can have beneficial effects on disease activity by positively modulating the intestinal epithelial lining.

## 1. Introduction

Inflammatory bowel disease (IBD), consisting of Crohn’s disease (CD) and ulcerative colitis (UC), is a chronic debilitating disease. With a mean incidence peak between 15 and 25 years, patients are affected in their reproductive years [1,2], and concerns regarding pregnancy are therefore common in this patient group. Pregnancy is accompanied by hormonal, immunological and microbial changes, allowing the growth of an allogeneic fetus. In light of these physiological changes, a disease modulatory role for pregnancy has been speculated upon, and the effect of pregnancy on the course of IBD has been a topic of research for many years. However, studies have shown contradicting data. Castiglione et al. reported fewer disease relapses in the three years post-partum than before pregnancy in both CD and UC patients [3], suggesting a positive effect of pregnancy on disease parameters. A 10-year follow-up study confirmed this in both UC (from 0.34 to 0.18 relapses/year) and CD patients (from 0.76 to 0.12 relapses/year) [4]. In addition, it appears safe to stop anti-TNFα treatment in pregnant IBD patients without increasing the risk of relapses [5,6]. In contrast, Pedersen et al. reported a higher relapse rate (RR) during pregnancy (RR 2.19) and postpartum (RR 6.22) in pregnant UC patients than in non-pregnant UC patients, whereas the relapse rates were similar for pregnant and non-pregnant CD patients [7].

During pregnancy, the sex hormones estrogen and progesterone levels steadily increase until the third trimester. Several studies have suggested that estrogen and progesterone have anti-inflammatory immune-modulatory effects by reducing inflammatory cytokines [8,9,10]. Nevertheless, the direct effect of pregnancy hormones on IBD remains unclear. Khalili et al. showed that postmenopausal women who use oral contraceptives have a higher risk of developing mainly UC [11], which was further supported in a meta-analysis showing that the risk of developing CD increased with exposure to oral contraceptives, but normalized when contraceptives were stopped [12]. In contrast, Kane et al. described a protective effect of estrogen on the bowel in women with IBD [13]. Animal studies on the effect of pregnancy hormones on IBD have also shown conflicting data. HLA-B27 transgenic rats with chronic diarrhea had better stool scores after treatment with 17α-ethynyl-17β-estradiol for 5 days [14]. Another animal study found an anti-inflammatory effect of a supraphysiological dose of 17β-estradiol in dextran sodium sulfate (DSS) murine model for colitis, but a pro-inflammatory effect in a dinitrobenzene sulfonic acid (DNB) colitis model [15], suggesting that the effect of estradiol on bowel symptoms may be context dependent. In rats with trinitrobenzene sulphonic acid (TNBS)-induced colitis, progesterone also significantly decreased oxidative damage in the colonic mucosa [16].

While immunological parameters have been extensively studied in the context of pregnancy, an underexplored avenue of investigation is the direct effect of estrogen and progesterone on the epithelial barrier. This barrier is the intestine’s first line of defense against invading bacteria. In IBD patients, the epithelial lining is weakened and, therefore, less resistant to pathogens. This “leaky gut” process is mainly caused by immune cells producing inflammatory cytokines, such as IFNɣ an TNFα [17,18] and is worsened during active disease, when there is an additional reduction in tight junctions which regulate epithelial permeability [19]. Furthermore, inflammatory triggers, such as cytokines and nitric oxide, can impede protein folding in IBD. This can lead to accumulation of unfolded proteins inside the endoplasmic reticulum (ER), which results in an unfolded protein response and ER stress. The gut epithelium expresses receptors for both estrogen (ERα and β) and progesterone [20] and animal studies have shown an increase in the transmembrane protein occludin in response to estrogen, leading to improved intestinal epithelial barrier function [21]. Moreover, the administration of estrogen or blockage of testosterone in male rats resulted in gut epithelial cells being more resistant to inflammation [22]. Thus, while there is some evidence suggesting a modulatory role of sex hormones on intestinal barrier functions, it is as yet unclear whether these hormones—levels of which increase during pregnancy—directly affect epithelial barrier cells. Here, we employed human colonic adenocarcinoma cell lines (Caco2, HCT116, T84) and organoids from human colon biopsies as model systems to study epithelial barrier function, production of cytokines, and ER stress modulation in response to sex hormones.

## 2. Methods

### 2.1. Cell Lines

Colorectal epithelial cell lines Caco2, HCT116 and T84 were cultured in Dulbecco’s Modified Eagles Medium (DMEM, Lonza, Basel, Switzerland) supplemented with 100 U/mL penicillin, 100 mg/mL streptomycin (Life technologies, Bleiswijk, NL, USA) and 10% fecal calf serum (FCS, Sigma-Aldrich, St. Louis, MO, USA). Cells were maintained at 37 °C in a 5% CO_2_ humidified setting.

### 2.2. Organoid Culture

Non-inflamed intestinal biopsies were collected from three female UC patients undergoing endoscopy for evaluation of their disease. In two of these patients, no inflammation was observed, and organoids were generated from non-inflamed colonic biopsies. A third patient demonstrated endoscopic disease activity in the colon, and biopsies obtained from the lesion as well as biopsies obtained in a non-inflamed section of the colon were obtained for a paired comparison. Organoids were obtained as previously described [23]. Biopsies were collected in PBS and transferred into a 15 mL tube containing 10 mL complete chelating solution (CCS, MilliQ H_2_O was supplemented with 1.0 g/L of Na2HPO4-2H2O, 1.08 g/L of KH2PO4, 5.6 g/L of NaCl, 0.12 g/L of KCl, 15 g/L of Sucrose, 10 g/L of D-Sorbitol and 80 lg/L of DL-dithiothreitol). Crypts were isolated by adding 100 uL 0.5 M EDTA for 35 min at 4 °C followed by mechanical disruption of biopsies. Supernatant with crypts was treated with FCS and crypts were re-suspended in 12 mL cold advanced DMEM (Advanced DMEM/F12, 5 mL 100x GlutaMAX (GMX), 1% P/S, 500 μL Gentamicin and 5 mL 1 M HEPES). After centrifugation, crypts were suspended in 50 μL growth factor reduced phenol-red free Matrigel (Corning, Bedford, MA, USA). Then, a 50 μL droplet of Matrigel/crypt mix was placed in the center of each well of a 24-well plate, and was subsequently incubated at 37 °C with 5% CO2 for 15 min. A total of 700 μL of culture medium was added per well. The culture medium was supplemented with CMGF-, 2% of B-27 supplements (Gibco, Grand Island, NE, USA), 1% of N2 Supplements (Gibco, Grand Island, USA), 500 pg/L of EGF, 1 mM n-Acetyl Cysteine, 10 mM Nicotinamide, 0.5 μM A83-01 (TGF-b inhibitor), 3 μM SB202190 (p38 inhibitor), 20% (*v/v*) of R-Spondin 1 (conditioned medium), 10% (*v/v*) of Noggin (conditioned medium) and 50% (*v/v*) of Wnt3a (conditioned medium). The culture medium was refreshed every 3 days, and organoids were passaged every 6–7 days. Each well contained 10 or more organoids.

### 2.3. Reverse Transcriptase Polymerase Chain Reaction (rt-PCR)

We used rt-PCR to validate estrogen-beta and progesterone receptor expression on the intestinal cell lines. Ribosomal protein (*RP2*) primers were used as control [24]. RNA was isolated using a NucleoSpin^®^ RNA kit (MACHEREY-NAGEL, Düren, Germany) and cDNA was synthesized using the TAKARA reverse transcription system (TAKARA BIO INC, Shiga, Japan). PCR was performed in a 25 μL reaction, using GoTaq polymerase and GoTaq Flexi buffer, 2 mM MgCl_2_ (Promega, Madison, WI, USA), dNTP (0.5 mM each, Roche, Basel, Switzerland), 50 ng template and 0.5 nM primer (for primers sequences see Supplementary Table S1). Quantitative PCR (QPCR) was used to determine *claudin 1*, *claudin 2*, *zonula occludens 1* (*ZO-1*) and *occludin* expression. *HPRT1* primers were used as control. For each sample 10 uL SYBR^TM^ Select Master Mix and 0.5 nM primer was used. All experiments were performed a minimum of 3 times.

### 2.4. Scratch Assay

Scratch assays were performed on Caco2 and HCT116 cell lines as described previously [25]. In short, cell monolayers were scratched with a pipette tip, washed twice, and treated with 1 μM estrogen and/or progesterone. Photographs were taken (Axiovert200 M microscope; Carl Zeiss BV, Sliedrecht, The Netherlands) to analyze the percentage of open wound area at 24 h (ImageJ software; US National Institutes of Health, Bethesda, MD, USA). Five independent wells were analyzed per condition, with two measure-sites per scratch.

### 2.5. MTT

Cell viability was assessed using MTT assays as described previously [26]. Cells were treated with estrogen and/or progesterone (Sigma Aldrich, St Louis, MA, USA). After 24 h, 48 h and 72 h, cells were incubated with 5 mM MTT (3-(4,5-Dimethylthiazol-2-yl)-2,5-diphenyltetrazolium bromide, Sigma Aldrich, St Louis, MA, USA) for 3 h and colorimetric changes were measured using a microplate reader (Model 680XR Bio-Rad, Hercules, CA, USA) at 490 and 595 nm. A minimum of three independent experiments were performed with each measurement in performed in duplicate.

### 2.6. TEER

Transepithelial resistance was measured using the Epithelial Voltohmmeter (EVOM2, Sarasota, FL, USA). Caco2 and T84 cells were seeded in a Transwell (6,5 mm insert, Costar, Kennebunk, ME, USA) and grown to confluency. Cells were subsequently stimulated with 10 µM estrogen and/or progesterone and resistance was measured at 0, 24 and 72 h. A minimum of three independent measurements were performed for time point.

### 2.7. Enzyme Linked Immunosorbent Assay (ELISA)

Caco2 cells were plated at 900,000 per well in 24-well plates. Upon attachment to the plate, cells were treated as described in the text and supernatant was harvested after 24 h. Experiments with cells were performed four times and experiments with organoids were performed nine times. Cytokine levels in supernatants from intestinal cells and organoids were determined by ELISA (Ready-SET-Go!^®^ eBioscience, San Diego, CA, USA) as per manufacturer’s instructions. All samples were tested in duplicate in the ELISAs.

### 2.8. Western Blotting

Caco2, HCT116 cells and organoids were treated with tunicamycin (0.5μM) in the presence or absence of 10 μM estrogen and/or progesterone. Cells were lysed in Laemmli buffer (100 mM Tris–HCl (pH 6.8), 200 mM dithiothreitol, 4% SDS, 0.1% bromophenol blue, 20% glycerol, and 2% DTT) and proteins were resolved by SDS-PAGE and transferred to polyvinylidene difluoride membranes (Merck chemicals BV, Amsterdam, the Netherlands) as described [27]. Membranes were blocked in 50% odyssey blocking buffer (LI-COR Biosciences, Lincoln, NE, USA) in PBS/0.05% Tween-20 and incubated overnight at 4 °C with primary antibody. After washing in PBS-Tween, membranes were incubated with IRDye^®^ antibodies (LI-COR Biosciences, Lincoln, NE, USA) for 1 h. Detection was performed using an Odyssey reader and analyzed using manufacturer’s software. All experiments were performed a minimum of three times.

### 2.9. Statistical Analysis

For in vitro and ex vivo experiments, normality of distribution was assessed with D’Agostino and Pearson Omnibus normality test. When passing normality test or when there were insufficient numbers to calculate normality, parametric testing was performed; otherwise, non-parametric tests were employed. Student’s *t*-tests were performed for comparisons of two groups. Mann-Whitney U tests were used for non-parametric data. For all tests, one or two-sided (as appropriate) *p*-values < 0.05 were considered statistically significant. Graphs show mean ± SEM or median with IQR. Analyses were performed using Graphpad Prism version 5.01 (San Diego, CA, USA).

## 3. Results

### 3.1. Estrogen and Progesterone Stimulate Wound Healing in Intestinal Epithelium

**Figure 1:**
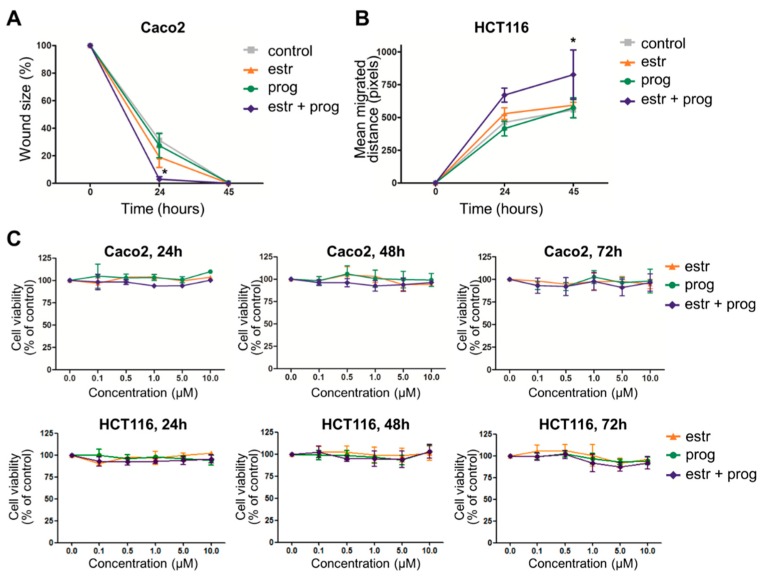
Estrogen and progesterone improve epithelial barrier healing. Caco2 cells’ (**A**) and HCT116 cells’ (**B**) confluent layers were scratched and cultured in the absence or presence of estrogen and/or progesterone. Cells were photographed at 0, 24 and 45 h. Scratch assay of CACO2 cells is presented as mean percentage wound size. Scratch assay of HCT116 is presented as mean migration of wound edges in pixels. Results of five independent experiments are shown, with a minimum of two scratches per stimulus. (**C**) MTT assay shows that sex hormones do not affect viable cell numbers (concentrations of hormone treatment indicated on the *X* axis). Measurements were done in three independent experiments at *t* = 24, 48 and 72 h. * statistical significant (*p* < 0.05).

One of the treatment goals in IBD is achievement of mucosal healing, as this is associated with reduced relapse rates and better quality of life [28]. We therefore first investigated the effect of sex hormones on wound healing of epithelial barriers. After confirming the expression of progesterone and estrogen receptor in epithelial cells on mRNA level (Supplementary Figure S1), 2D layers of Caco2 and HCT116 cells were scratched and the wound closure was measured in the presence or absence of progesterone and/or estrogen. Both the size of the wound, and the migrated distance of the wound-edges were determined. We found that when treated with both estrogen and progesterone, Caco2 cells showed a faster reduction in wound size than unstimulated cells (*p* = 0.006 at *t* = 24, Figure 1A), resulting in complete wound closure after 24 h. In the less motile HCT116 cell line, complete wound healing was not achieved within this timeframe, but there was a faster migration of cells when stimulated with sex hormones (*p* = 0.044 at *t* = 24, Figure 1B). As a faster closure of wound area could conceivably also be achieved by increased proliferation, we next investigated the number of viable cells in the absence and presence of sex hormones. Figure 1C shows that neither progesterone nor estrogen or the combination thereof substantially affects Caco2 or HCT116 cell growth. Together, these findings imply a positive modulatory role for sex hormones on epithelial wound healing.

### 3.2. Estrogen and Progesterone Alleviate ER Stress in Intestinal Epithelium

**Figure 2:**
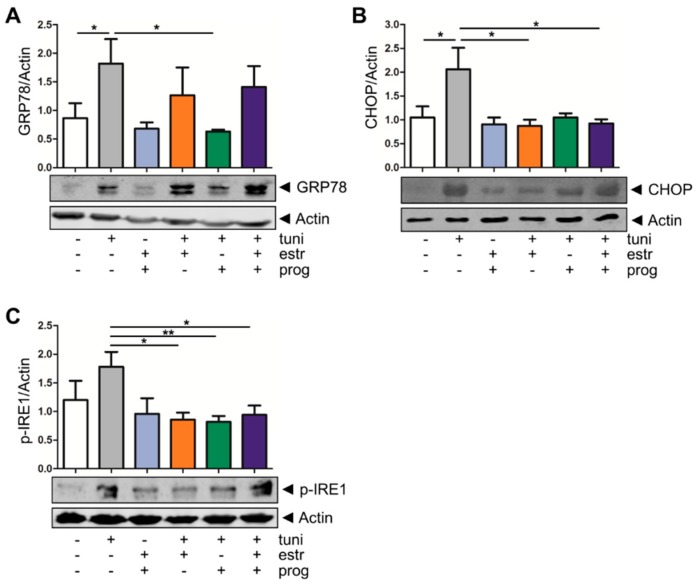
Sex hormones reduce endoplasmic reticulum (ER) stress in inflammatory bowel disease (IBD) organoids. Western blot analysis of the ER stress markers GRP78 (**A**) CHOP (**B**) and p-IRE1 (**C**) in organoids. Induction of ER stress by stimulation with tunicamycin for 20 h results in an upregulation GRP78 protein expression. Four hours of stimulation with 10 nM tunicamycin resulted in an upregulation of CHOP protein expression and 6 h of stimulation with 10 nM tunicamycin resulted in an upregulation of the p-IRE1 protein expression. Addition of estrogen or progesterone attenuates ER stress induction. Upper panels show mean densitometry values of the ER stress proteins, corrected for actin levels in the same lanes, of five independent experiments; lower panels show representative examples of the blots. */** statistical significant (*p* < 0.05).

Next, we determined whether sex hormones may affect ER stress in intestinal barrier cells. We obtained inflamed and non-inflamed intestinal tissue biopsies from one UC patient in order to compare these two biopsies but did not observe differences in the IL-8 cytokine production, nor did we find significant differences in *claudin 1*, *claudin 2*, *occludin* or *ZO-1* expression (Supplementary Figure S2A–D). These results are in line with those of other studies, showing a loss of the inflammatory signature in ex vivo cultured organoids [29]. To this end, we mimicked inflammation-induced cellular protein folding defects by stimulating human colonic organoids with tunicamycin, which resulted in upregulation of the ER stress markers GRP78 (BiP) and its downstream target CHOP, as well as phosphorylation of IRE1, a kinase which autophosphorylates upon cellular ER stress [30] (Figure 2). Thus, tunicamycin stimulation of organoids can serve as a model for epithelial barrier stress. Upon addition of sex hormones, ER stress induction as determined by GRP78 expression was attenuated, in particular in the case of progesterone (1.819 ± 0.4293 to 0.6333 ± 0.0333, *p* = 0.0420, Figure 2A). CHOP expression was also reduced upon co-treatment of tunicamycin-treated organoids with either estrogen alone (2.062 ± 0.4497 to 0.8741 ± 0.1292, *p* = 0.0147) or in combination with progesterone (2.062 ± 0.4497 to 0.9250 ± 0.08539, *p* = 0.0397, Figure 2B). Lastly, we investigated IRE1, and showed that in our organoid model IRE-1 phosphorylation levels significantly decreased when estrogen (1.780 ± 0.2615 to 0.8600 ± 0.1208, *p* = 0.0127), progesterone (1.780 ± 0.2615 to 0.8200 ± 0.1020, *p* = 0.0091) or the combination thereof were added (1.780 ± 0.2615 to 0.9400 ± 0.1631, *p* = 0.0260, Figure 2C). We validated the stress relieving function of the sex hormones in the epithelial barrier models HCT116 and Caco2, again demonstrating that tumicamycin-induced GRP78 levels (Supplementary Figure S3A,B) and IRE1 phosphorylation (Supplementary Figure S3C) are reduced upon treatment of epithelial cells with progesterone, estrogen or the combination thereof. Thus, the data demonstrate that sex hormones may protect epithelial barrier cells from damage inflicted by ER stress.

### 3.3. Pro-Inflammatory Cytokine Production by Intestinal Epithelial Cells Is Decreased in the Presence of Progesterone and Estrogen

**Figure 3:**
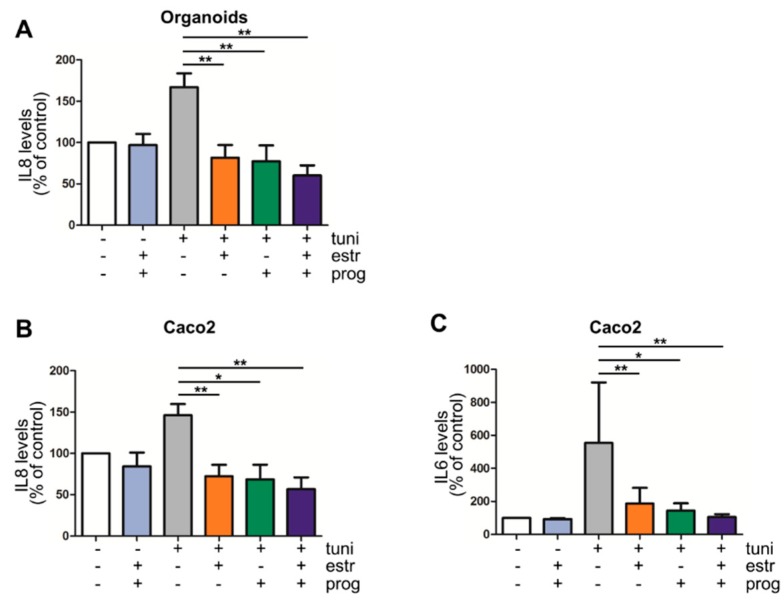
Progesterone and estrogen reduce IL6 and IL8 secretion in IBD inflammation models. Estrogen and progesterone significantly reduce IL8 production in an inflammation organoid model of IBD (**A**) (n = 9) as well as the Caco2 cell line model (**B**) (n = 4). IL6 production in Caco2 cells was also decreased by sex hormones (**C**) (n = 4). */** statistical significant (*p* < 0.05).

Deregulation of pro-and anti-inflammatory cytokines and interleukins is seen in IBD. In particular IL8, a neutrophil chemoattractant, and IL6, involved in perpetuating the immune reaction, are found in increased quantities in inflamed mucosa [31]. We investigated these cytokines in our cell culture models. Excretion of IL8 and IL6 was low in resting organoids and Caco2 cells, and thus not modulated by progesterone and estrogen (Figure 3A–C). However, treatment of cells with tunicamycin enhanced the levels of these pro-inflammatory cytokines, further demonstrating the pro-inflammatory effect of ER stress induction in these model systems. Next, we tested whether estrogen and progesterone influence this stress-induced cytokine production by organoids and Caco2 cells. As shown in Figure 3A, IL8 production in tunicamycin-treated organoids was significantly reduced when stimulated with estrogen, progesterone and both (*p* = 0.0040 vs. *p* = 0.0078 vs. *p* = 0.0040 respectively, Figure 3A), which was also seen in Caco2 cells (*p* = 0.0092 vs. *p* = 0.0138 vs. *p* = 0.0040 respectively, Figure 3B). Furthermore, when Caco2 cells co-stimulated with estrogen, progesterone and both sex hormones a decrease of IL6 cytokine was measured (*p* = 0.0093 vs. *p* = 0.0125 vs. *p* = 0.0021 respectively, Figure 3C). IL6 was not detected in organoids cultures, nor were TNFα or IL10.

### 3.4. Improved Barrier Function Strength in the Presence of Estrogen and Progesterone

**Figure 4:**
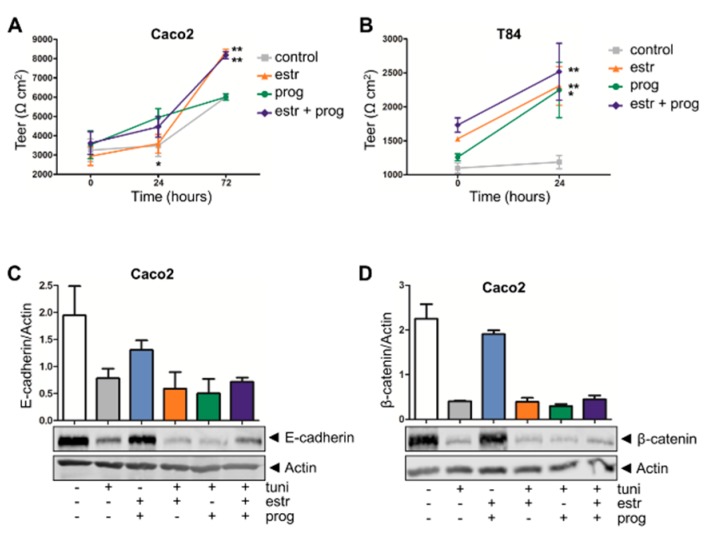
Sex hormones improve epithelial barrier function. Transepithelial electrical resistance (TEER) measurement of Caco2 (**A**) and T84 cell lines (**B**) show an increase in resistance when stimulated with sex hormones. Results of five independent experiments are shown. (**C**,**D**) Western blot analysis of the adhesion junction molecules E-cadherin (**C**) and β-Catenin (**D**). Neither constitutive levels nor stress-induced decreases in E-cadherin or β-Catenin levels are modulated by sex hormones. Upper panels show densitometry values (corrected for actin levels in the same lane) of two independent experiments; lower panels show representative examples. */** statistical significant (*p* < 0.05).

In IBD, weakening of the intestinal lining can result in a decreased resistance to pathogens. For the determination of the integrity of this epithelial barrier, we measured the transepithelial electrical resistance (TEER). Compared to controls, in Caco2 cells the barrier function was increased by estrogen (6007 ± 105 to 8347 ± 140, *p* = 0.0002, *t* = 72 h, Figure 4A) as well as the combination of progesterone and estrogen (6007 ± 105 to 8177 ± 180, *p* = 0.0005, *t* = 72 h, Figure 4A), although this latter combination treatment was not more efficient when compared to estrogen alone. As the growth pattern of HCT116 cells does not result in a confluent cell layer allowing TEER measurements, we further validated these results in another intestinal epithelial cell line, T84, which does form a resistant monolayer. As for Caco2 cells, barrier function was improved in T84 cells stimulated with sex hormones for 24 h. Both estrogen and progesterone alone increased TEER in these cell layers (neg vs. estrogen 1186 ± 97 to 2307 ± 284, *p* = 0.0022; neg vs. progesterone 1186 ± 97 to 2247 ± 407, *p* = 0.0195), and the combination treatment also improved barrier strength (neg vs. estrogen + progestorone, 1186 ± 97 to 2516 ± 418, *p* = 0.0045, Figure 4B).

**Figure 5:**
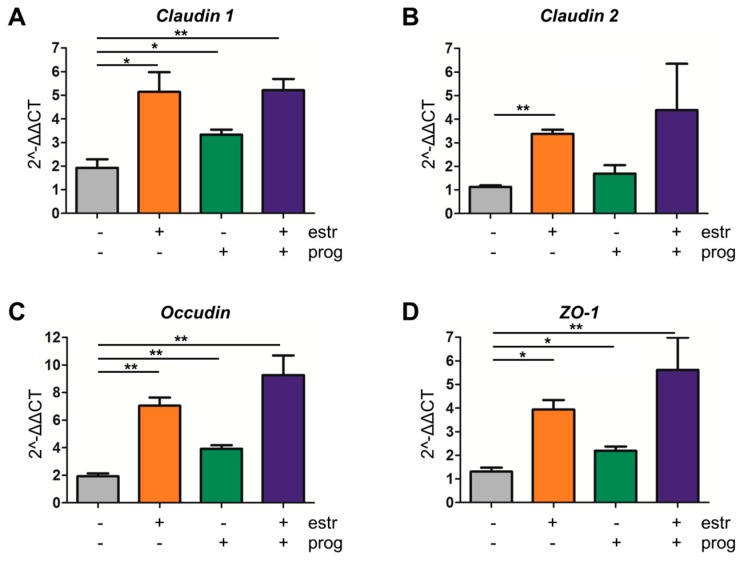
Improvement of tight junction dynamics by sex hormones. Quantitative PCR (QPCR) analysis of the tight junction *claudin 1* (**A**), *claudin 2* (**B**), *ZO-1* (**C**) and *occludin* (**D**) were measured in the IBD organoid inflammation model. Co-stimulation with estrogen, progesterone, or a combination resulted in upregulation of *claudin 1*, *ZO-1* and *occludin* (three independent experiments). For *claudin 2* this was only for co-stimulation with estrogen (five independent experiments). */** statistical significant (*p* < 0.05).

In an effort to clarify the molecular mechanisms contributing to improved barrier function upon sex hormone treatment, we first determined protein levels of E-cadherin and β-catenin, two adherent junction proteins modulating epithelial cell–cell adhesion [32]. However, treatment with a combination of progesterone and estrogen did not modulate expression levels of these proteins (see Figure 4C,D for Caco2 cells, Supplementary Figure S5A,B for HCT116 cells). As a control, we showed that stress induction in these epithelial cells with tunicamycin reduced the expression of E-cadherin or β-catenin, but again this was not modulated by sex hormones (Figure 4C,D, Supplementary Figure S5A,B). Next, we investigated whether an improvement in tight junction dynamics could underlie the positive effect of the sex hormones on barrier integrity. QPCR analysis showed that in our organoid inflammation model *claudin 1* expression was significantly upregulated upon treatment with either estrogen, progesterone, or both (*p* = 0.0239, *p* = 0.0280 and *p* = 0.0053, respectively, Figure 5A). Similarly, mRNA levels of and *occludin* (*p* = 0.0013, *p* = 0.0034 and *p* = 0.0069, respectively, Figure 5C) and *ZO-1* (*p* = 0.0040, *p* = 0.0242, *p* = 0.0359, respectively, Figure 5D) increased upon treatment with either sex hormone alone or the combination of both. For *claudin 2*, significant modulation was only observed with estrogen alone (*p* ≤ 0.0001, Figure 5B). Caco2 and HCT116 cells showed a similar tendency to upregulate *claudin 2* and *ZO-1* upon sex hormone treatment (Supplementary Figure S4A,B). Thus, while estrogen and progesterone positively modulate the barrier lining, the molecular mechanisms involved do not appear to include the E-cadherin/ β-catenin complex, but affects tight-junction dynamics.

## 4. Discussion

Several (auto)immune diseases, most noticeably rheumatoid arthritis and multiple sclerosis, have been shown to improve during pregnancy, a phenomenon that is generally ascribed to modulation of immunological parameters in response to rising progesterone and estrogen levels [33,34,35]. However, pregnancy can induce a range of physiological changes, including modulation of the gastrointestinal microbiome as well modulation of the gastrointestinal smooth musculature [36]. The complex disease etiology of IBD involves an altered immune response towards the intestinal microbiome in genetically susceptible individuals. Thus, with all these changes during pregnancy occurring in processes that affect IBD pathophysiology, a beneficial effect of pregnancy in IBD has been speculated upon [37]. An important aspect of IBD pathophysiology includes a weakened intestinal barrier function, yet the direct consequences of pregnancy hormones on intestinal barrier cells thus far have remained unclear. Here, we show that progesterone and estrogen can directly improve epithelial barrier functions, suggesting a positive modulatory effect of these hormones on the intestinal epithelial lining during pregnancy.

Firstly, our results showed that estrogen and progesterone, without affecting cell proliferation, improved wound healing and epithelial barrier strength. These results are in line with those of Braniste et al., who concluded that there is a physiological link between estrous cycle-dependent changes in hormone levels and intestinal permeability changes, and also demonstrated an ERβ-mediated increase of *occludin* in epithelial cells in the colon [21]. We demonstrate here that this also applies for *claudin 1*, *claudin 2* and *ZO-1*. Secondly, we show that estrogen and progesterone can alleviate ER stress in intestinal epithelial cells. We and others have previously suggested that mucosal ER stress is linked to the development of IBD [38,39,40]. ER stress is known to induce inflammatory responses, and contributes to a rise in pro-inflammatory cytokines in several cell types [41]. Here, we demonstrate that induction of ER stress also increases IL8 and IL6 levels in our epithelial models, including IBD organoids, and we thus employed induction of ER stress as a model of inflammation in these systems. Several groups have now cultured organoids from IBD patients, and these cultures provide a good model to investigate the barrier function in an IBD-specific context [42]. However, while some patient- and barrier-specific transcription patterns and functions are present in these in vitro cultures, other studies have also suggested that inflammation in these organoid systems is not propagated, and may be best modelled by addition of exogenous stimulants [43], as inflammatory phenotype might be lost upon culture of organoids beyond four weeks [29]. Our data suggest that estrogen and progesterone may reduce inflammation-induced pro-inflammatory cytokine production by epithelial barrier cells. Both IL6 and IL8 Levels are increased in the inflamed mucosa from IBD patients [44,45], and our own observations suggest that pro-inflammatory cytokine levels (e.g., IL6, IL8, TNFα) decrease significantly in IBD patients upon conception (unpublished data). Our current study suggests that this might be partly due to a direct effect of pregnancy hormones on cytokine production in epithelial cells. A study showing significantly lower systemic plasma cytokine levels of IL6, IL8 and IL10 in premenopausal women than in men corroborates this notion [46].

To our knowledge, this is the first study reporting on the direct effect of pregnancy hormones on different aspects of the intestinal epithelial barrier. One limitation of this study is that we employed one concentration of the sex hormones. During pregnancy both estrogen and progesterone increase, but hormone levels in vitro are not one-on-one comparable with hormone levels in vivo, and the modulation of levels of these steroid hormones in the mucosal barrier are as yet unknown. Thus, it is conceivable that we may have used supra physiological levels when adding 10 μM estrogen and progesterone as a stimulus in vitro. We used equimolar concentrations of the hormones for a fair comparison between these hormones. Our results imply that both progesterone and estrogen have beneficial effects on epithelial barrier functions, with additive effects on most processes studied here, which allows statistical significance to be reached while not reaching this significance with one or both of the single treatments. Another limitation of this study is that the effect of IBD medication is not taken into account in these in vitro models. However, our own unpublished data suggest that IBD medication does not affect cytokine production in pregnant IBD patients. 5-ASA and azathioprine are considered of low risk during pregnancy and therefore often used in clinical practice Studies investigating the effect of these treatments on the epithelial barrier function have indicated a positive effect in an intestinal organoids model [47]. Furthermore, 5-ASA was able to inhibit IFN-gamma induced impairment of the epithelial barrier [48]. Thus, overall the effect seems to be positive rather than negative and suggests that there might be an additional positive effect of some IBD medication on the epithelial barrier. Nevertheless, our study provides additional insight into the mechanism of action of estrogen and progesterone on the intestinal epithelial lining and supports a protective role of pregnancy hormones on epithelial barrier function during pregnancy.

## Supplementary Materials

The supplementary materials are available online at https://www.mdpi.com/2073-4409/8/3/261/s1.

## References

[1] Kaplan G.G. (2015). The global burden of IBD: From 2015 to 2025. Nat. Rev. Gastroenterol. Hepatol..

[2] Johnston R.D., Logan R.F.A. (2008). What is the peak age for onset of IBD?. Inflamm. Bowel. Dis..

[3] Castiglione F. (1996). Effect of pregnancy on the clinical course of a cohort of women with IBD. Ital. J. Gastroenterol..

[4] Riis L., Vind I., Politi P., Wolters F., Vermeire S., Tsianos E., Freitas J., Mouzas I., Ruiz Ochoa V., O’Morain C. (2006). Does pregnancy change the disease course? A study in a European cohort of patients with inflammatory bowel disease. Am. J. Gastroenterol..

[5] de Lima A., Zelinkova Z., van der Ent C., Steegers E.A.P., van der Woude C.J. (2016). Tailored anti-TNF therapy during pregnancy in patients with IBD: Maternal and fetal safety. Gut.

[6] Zelinkova Z., van der Ent C., Bruin K.F., van Baalen O., Vermeulen H.G., Smalbraak H.J.T., Ouwendijk R.J., Hoek A.C., van der Werf S.D., Kuipers E.J. (2013). Effects of discontinuing anti-tumor necrosis factor therapy during pregnancy on the course of inflammatory bowel disease and neonatal exposure. Clin. Gastroenterol. Hepatol..

[7] Pedersen N., Bortoli A., Duricova D., Dinca R., Panelli M.R., Gisbert J.P., Zoli G., López-Sanromán A., Castiglione F., Riegler G. (2013). The course of inflammatory bowel disease during pregnancy and postpartum: A prospective European ECCO-EpiCom Study of 209 pregnant women. Aliment. Pharmacol. Ther..

[8] Lei B., Mace B., Dawson H.N., Warner D.S., Laskowitz D.T., James M.L. (2014). Anti-Inflammatory Effects of Progesterone in Lipopolysaccharide-Stimulated BV-2 Microglia. PLoS ONE.

[9] Straub R.H. (2007). The Complex Role of Estrogens in Inflammation. Endocr. Rev..

[10] Preciado-Martínez E., García-Ruíz G., Flores-Espinosa P., Bermejo-Martínez L., Espejel-Nuñez A., Estrada-Gutiérrez G., Razo-Aguilera G., Granados-Cepeda M., Helguera-Repetto A.C., Irles C. (2018). Progesterone suppresses the lipopolysaccharide-induced pro-inflammatory response in primary mononuclear cells isolated from human placental blood. Immunol. Investig..

[11] Khalili H., Higuchi L.M., Ananthakrishnan A.N., Manson J.E., Feskanich D., Richter J.M., Fuchs C.S., Chan A.T. (2012). Hormone Therapy Increases Risk of Ulcerative Colitis but not Crohn’s Disease. Gastroenterology.

[12] Cornish J.A., Tan E., Simillis C., Clark S.K., Teare J., Tekkis P.P. (2008). The Risk of Oral Contraceptives in the Etiology of Inflammatory Bowel Disease: A Meta-Analysis. Am. J. Gastroenterol..

[13] Kane S.V., Reddy D. (2008). Hormonal Replacement Therapy After Menopause Is Protective of Disease Activity in Women with Inflammatory Bowel Disease. Am. J. Gastroenterol..

[14] Harnish D.C., Albert L.M., Leathurby Y., Eckert A.M., Ciarletta A., Kasaian M., Keith J.C. (2004). Beneficial effects of estrogen treatment in the HLA-B27 transgenic rat model of inflammatory bowel disease. Am. J. Physiol. Liver Physiol..

[15] Verd E.F., Deng Y., Bercik P., Collins S.M. Modulatory Effects of Estrogen in Two Murine Models of Experimental Colitis. http://www.physiology.org/doi/pdf/10.1152/ajpgi.00460.2001.

[16] Karatepe O.I., Altiok M.I., Battal M., Kamali G.I., Kemik A.V., Aydin T.V., Karahan S. (2012). The effect of progesterone in the prevention of the chemically induced experimental colitis in rats Efeito da progesterona na prevenção de colite experimental induzida quimicamente em ratos. Acta Cirúrgica Bras.

[17] Bouma G., Strober W. (2003). The immunological and genetic basis of inflammatory bowel disease. Nat. Rev. Immunol..

[18] Shen L., Su L., Turner J.R. (2009). Mechanisms and functional implications of intestinal barrier defects. Dig. Dis..

[19] Gassler N., Rohr C., Schneider A., Kartenbeck J., Bach A., Obermüller N., Otto H.F., Autschbach F. (2001). Inflammatory bowel disease is associated with changes of enterocytic junctions. Am. J. Physiol. Liver Physiol..

[20] Konstantinopoulos P.A., Kominea A., Vandoros G., Sykiotis G.P., Andricopoulos P., Varakis I., Sotiropoulou-Bonikou G., Papavassiliou A.G. (2003). Oestrogen receptor beta (ERbeta) is abundantly expressed in normal colonic mucosa, but declines in colon adenocarcinoma paralleling the tumour’s dedifferentiation. Eur. J. Cancer.

[21] Braniste V., Leveque M., Buisson-Brenac C., Bueno L., Fioramonti J., Houdeau E. (2009). Oestradiol decreases colonic permeability through oestrogen receptor beta-mediated up-regulation of occludin and junctional adhesion molecule-A in epithelial cells. J. Physiol..

[22] Homma H., Hoy E., Xu D.-Z., Lu Q., Feinman R., Deitch E.A. (2005). The female intestine is more resistant than the male intestine to gut injury and inflammation when subjected to conditions associated with shock states. Am. J. Physiol. Liver Physiol..

[23] Dekkers J.F., Wiegerinck C.L., de Jonge H.R., Bronsveld I., Janssens H.M., de Winter-de Groot K.M., Brandsma A.M., de Jong N.W., Bijvelds M.J., Scholte B.J. (2013). A functional CFTR assay using primary cystic fibrosis intestinal organoids. Nat. Med..

[24] Yin Y., Wang Y., Dang W., Xu L., Su J., Zhou X., Wang W., Felczak K., van der Laan L.J., Pankiewicz K.W. (2016). Mycophenolic Acid Potently Inhibits Rotavirus Infection with a High Barrier to Resistance Development. https://ac.els-cdn.com/S0166354216301097/1-s2.0-S0166354216301097-main.pdf?_tid=6f7dfc30-12f2-43c0-82f3-2c4048172105&acdnat=1527171066_52bdb7527063bd4ac0e47a84685cc304.

[25] Lie M.R., van der Giessen J., Fuhler G.M., De Lima A., Peppelenbosch M.P., Van Der Ent C., van der Woude C.J. (2018). Low dose Naltrexone for induction of remission in inflammatory bowel disease patients. J. Transl. Med..

[26] Queiroz K., Ruela-De-Sousa R., Fuhler G., Aberson H., Ferreira C., Peppelenbosch M., Spek C.A. (2010). Hedgehog signaling maintains chemoresistance in myeloid leukemic cells. Oncogene.

[27] Somasundaram R., Nuij V.J., Van Der Woude C.J., Kuipers E.J., Peppelenbosch M.P., Fuhler G.M. (2013). Peripheral Neutrophil Functions and Cell Signalling in Crohs Disease. PLoS ONE.

[28] Peyrin-Biroulet L., Ferrante M., Magro F., Campbell S., Franchimont D., Fidder H. (2011). Results from the 2nd Scientific Workshop of the ECCO (I): Impact of mucosal healing on the course of inflammatory bowel disease. J. Crohn’s Colitis.

[29] Arnauts K., Verstockt B., Vancamelbeke M., Vermeire S., Verfaillie C., Ferrante M. Organoids Derived from Inflamed Intestinal Biopsies of Patients with Ulcerative Colitis Lose Their Inflammatory Phenotype during ex Vivo Culture. Preliminary Data Presented at the European Crohns and Colitis Organisation Meeting 2019. https://www.ecco-ibd.eu/publications/congress-abstract-s/abstracts-2019/item/op11-organoids-derived-from-inflamed-intestinal-biopsies-of-patients-with-ulcerative-colitis-lose-their-inflammatory-phenotype-during-italic-ex-vivo-italic-culture.html.

[30] Tschurtschenthaler M., Adolph T.E., Ashcroft J.W., Niederreiter L., Bharti R., Saveljeva S., Bhattacharyya J., Flak M.B., Shih D.Q., Fuhler G.M. (2017). Defective ATG16L1-mediated removal of IRE1α drives Crohn’s disease-like ileitis. J. Exp. Med..

[31] Powell N., Lo J.W., Biancheri P., Vossenkämper A., Pantazi E., Walker A.W., Stolarczyk E., Ammoscato F., Goldberg R., Scott P. (2015). Interleukin 6 Increases Production of Cytokines by Colonic Innate Lymphoid Cells in Mice and Patients with Chronic Intestinal Inflammation. Gastroenterology.

[32] Tian X., Liu Z., Niu B., Zhang J., Tan T.K., Lee S.R., Zhao Y., Harris D.C., Zheng G. (2011). E-cadherin/β-catenin complex and the epithelial barrier. J. Biomed. Biotechnol..

[33] Ysrraelit M.C., Correale J. (2019). Impact of sex hormones on immune function and multiple sclerosis development. Immunology.

[34] Barrett J.H., Brennan P., Fiddler M., Silman A.J. (1999). Does rheumatoid arthritis remit during pregnancy and relapse postpartum? Results from a nationwide study in the United Kingdom performed prospectively from late pregnancy. Arthritis Rheum.

[35] de Man Y.A., Dolhain R.J., van de Geijn F.E., Willemsen S.P., Hazes J.M. (2008). Disease activity of rheumatoid arthritis during pregnancy: Results from a nationwide prospective study. Arthritis Rheum.

[36] Soma-Pillay P., Nelson-Piercy C., Tolppanen H., Mebazaa A., Tolppanen H., Mebazaa A. (2016). Physiological changes in pregnancy. Cardiovasc. J. Afr..

[37] Konstantinov S.R., van der Woude C.J., Peppelenbosch M.P. (2013). Do pregnancy-related changes in the microbiome stimulate innate immunity?. Trends Mol. Med..

[38] Jostins L., Ripke S., Weersma R.K., Duerr R.H., McGovern D.P., Hui K.Y., Lee J.C., Schumm L.P., Sharma Y., Anderson C.A. (2012). Host-microbe interactions have shaped the genetic architecture of inflammatory bowel disease. Nature.

[39] Ishihara S., Aziz M.M., Yuki T., Kazumori H., Kinoshita Y. (2009). Inflammatory bowel disease: Review from the aspect of genetics. J. Gastroenterol..

[40] Deuring J.J., Fuhler G.M., Konstantinov S.R., Peppelenbosch M.P., Kuipers E.J., de Haar C., van der Woude C.J. (2014). Genomic ATG16L1 risk allele-restricted Paneth cell ER stress in quiescent Crohn’s disease. Gut.

[41] Kim S., Joe Y., Kim H.J., Kim Y.-S., Jeong S.O., Pae H.-O., Ryter S.W., Surh Y.J., Chung H.T. (2015). Endoplasmic reticulum stress-induced IRE1α activation mediates cross-talk of GSK-3β and XBP-1 to regulate inflammatory cytokine production. J. Immunol..

[42] Xu P., Becker H., Elizalde M., Masclee A., Jonkers D. (2018). Intestinal organoid culture model is a valuable system to study epithelial barrier function in IBD. Gut.

[43] Noben M., Verstockt B., de Bruyn M., Hendriks N., Van Assche G., Vermeire S., Verfaillie C., Ferrante M. (2017). Epithelial organoid cultures from patients with ulcerative colitis and Crohn’s disease: A truly long-term model to study the molecular basis for inflammatory bowel disease?. Gut.

[44] Grimm M.C., Elsbury S.K., Pavli P., Doe W.F. (1996). Interleukin 8: Cells of origin in inflammatory bowel disease. Gut.

[45] Mazzucchelli L., Hauser C., Zgraggen K., Wagner H., Hess M., Laissue J.A., Mueller C. (1994). Expression of interleukin-8 gene in inflammatory bowel disease is related to the histological grade of active inflammation. Am. J. Pathol..

[46] Frink M., Pape H.-C., Van Griensven M., Krettek C., Chaudry I.H., Hildebrand F. Influence of Sex and Age on Mods and Cytokines After Multiple Injuries. https://insights.ovid.com/pubmed?pmid=17224789.

[47] Khare V., Krnjic A., Frick A., Gmainer C., Asboth M., Jimenez K., Lang M., Baumgartner M., Evstatiev R., Gasche C. (2019). Mesalamine and azathioprine modulate junctional complexes and restore epithelial barrier function in intestinal inflammation. Sci. Rep..

[48] Di Paolo M.C., Merrett M.N., Crotty B., Jewell D.P. (1996). 5-Aminosalicylic acid inhibits the impaired epithelial barrier function induced by gamma interferon. Gut.

