# Supplementary material for: A Direct Effect of Sex Hormones on Epithelial Barrier Function in Inflammatory Bowel Disease Models
Source: Cells. 2019 Mar 19;8(3):261. doi: 10.3390/cells8030261 (PMC6468635; doi:10.3390/cells8030261)
Supplement: Supplementary file 1 [file cells-08-00261-s001.pdf]

Supplementary Materials

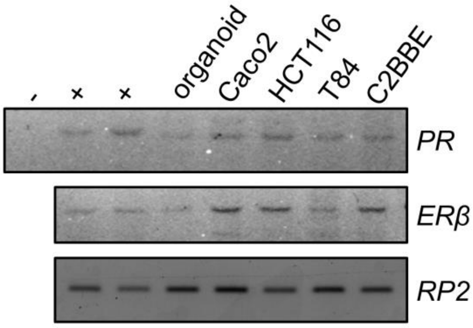

Figure S1. The presence of progesterone (PR) and estrogen-beta (ERβ) receptors mRNA was tested by PCR. Ribosomal protein 2 (*RP2*) was used as cDNA quality control. mRNA extracted from placental tissue was used as positive control (+), water was used as negative control (–) for the PCR.

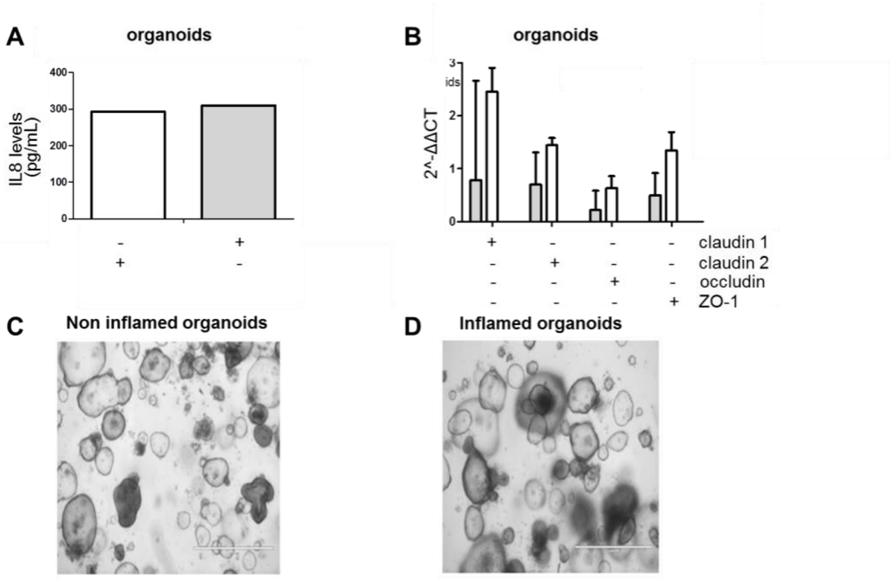

Figure S2. Comparison of organoids derived from inflamed vs. non-inflamed biopsies. ELISA analysis showed no differences in IL8 levels in inflamed vs. non-inflamed organoids **(A).** QPCR analysis showed no differences in *claudin 1, claudin 2, occludin* and *ZO-1* expression (**B**). Organoids derived from non-inflamed biopsies (**C**). Organoids derived from inflamed biopsies (D) were slower to grow, but did not show morphological differences.

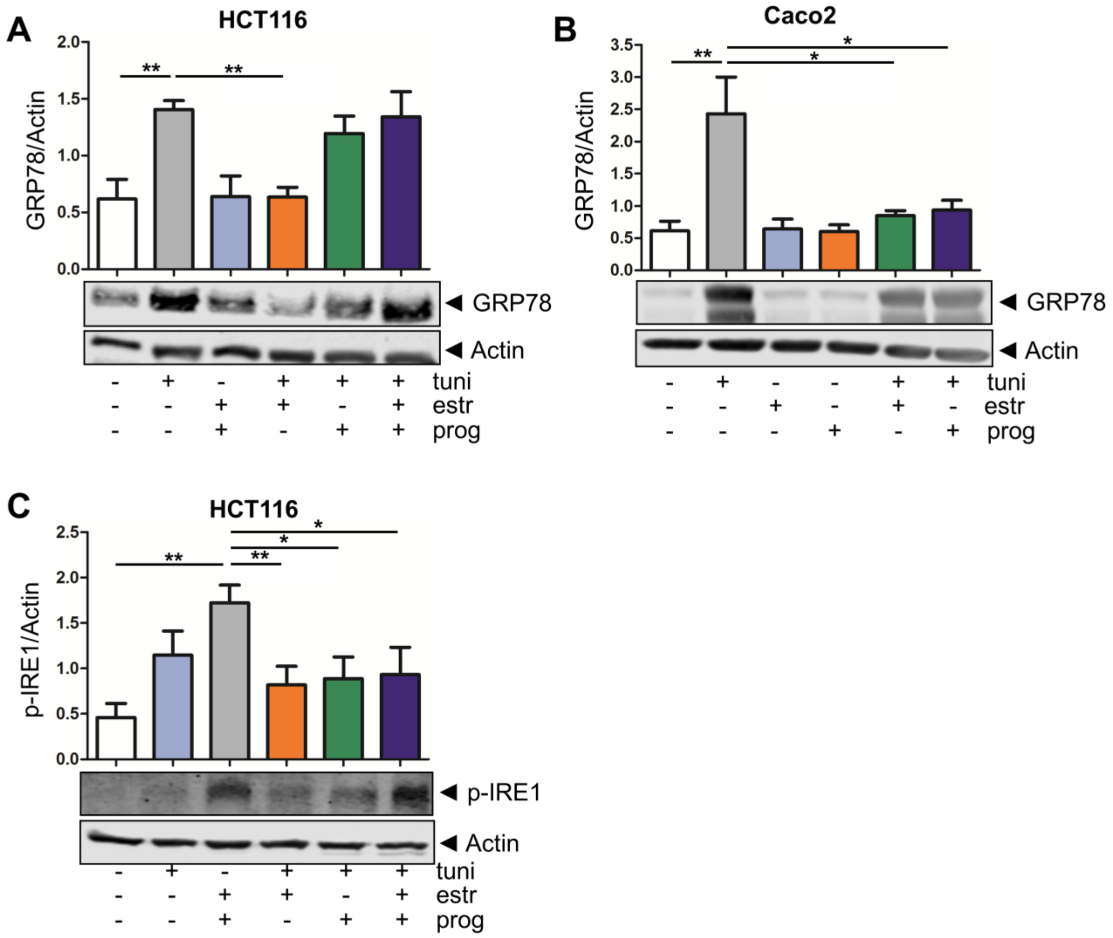

Figure S3. Sexhormones reduce ER stress in cell line. Sexhormones reduce ER stress in cell lines. Western blot analysis of the ER stress marker GRP78 in HCT116 (**A,** *n*=3) and Caco2 (**B,** *n*=6). Induction of ER stress by stimulation with tunicamycin for 20 h results in an upregulation GRP78 protein expression, which was decreased upon treatment with estrogen in HCT116 cells and progesterone or the combination of sexhormones in Caco2 cells. Similarly, phosphorylation of IRE1

in HCT116 cells (**C**) was reduced upon treatment with sexhormones (n=5). Upper panels show mean densitometry values of the ER stress proteins, corrected for actin levels in the same lanes, lower panels show representative examples of the blots.

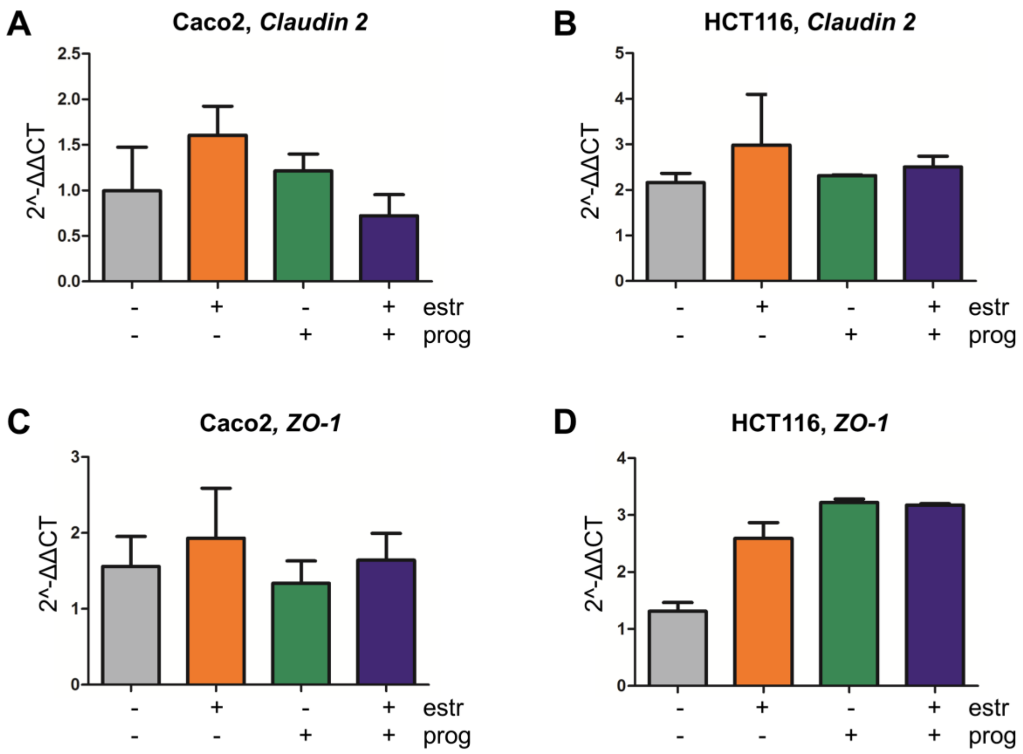

Figure S4. Improvement of tight junction dynamics. Improvement of tight junction dynamics. QPCR analysis of the tight junction *claudin 2* in Caco2 cells (**A**), *claudin 2* in HCT116 cells (**B**), *ZO-1* in Caco2 (**C**) and *ZO-1* in HCT116 (**D**). For CACO2, the results of four experiments are shown, for HCT116 of two experiments.

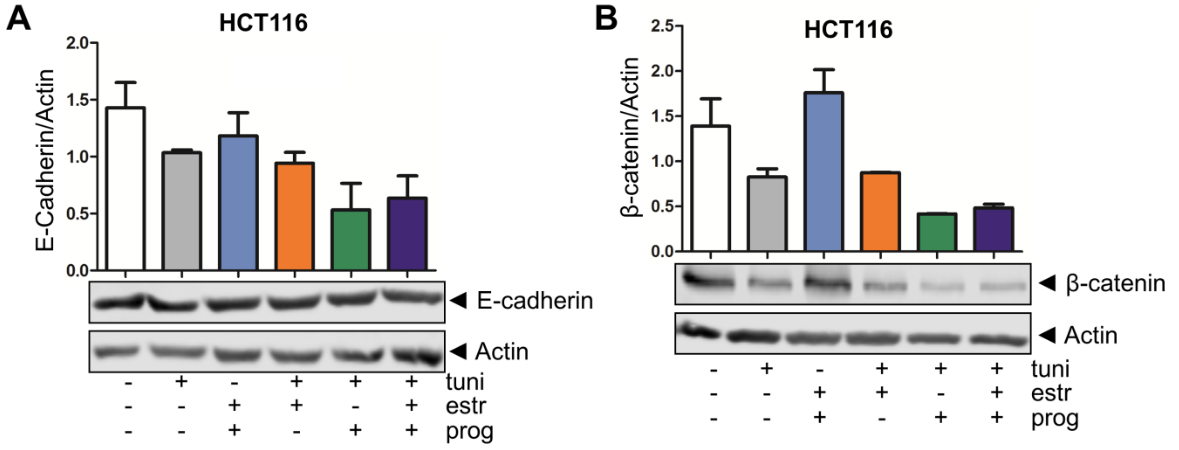

Figure S5. E-cadherin and beta-catenin levels in cell lines. Neither constitutive levels nor stress-induced decreases in E-cadherin or β-Catenin levels are modulated by sexhormones in HCT116 cells. Upper panels show densitometry values (corrected for actin levels in the same lane) of two independent experiments, lower panels show representative examples.

Table S1. Primer sequences

Estrogen receptor	forward: 5′-TGAAAAGGAAGGTTAGTGGGAACC,<br>Reverse: 5′-TGGTCAGGGACATCATCATGG
Progesteron receptor	forward: 5′-GATTCAGAAGCCAGCCAGAG,<br>Reverse: 5′-TGCCTCTCGCCTAGTTGATT
Claudin 1	forward: 5′-TGGTGGTTGGCATCCTCCTG-,<br>Reverse: 5′-AATTCGTACCTGGCATTGACTGG
Claudin 2	forward: 5′-GGCGGTAGCAGGTGGAGTC,<br>Reverse: 5′-CTTGGTAGGCATCGTAGTAGTTGG
ZO-1	forward: 5′-CAAGATAGTTTGGCAGCAAGAGATG,<br>Reverse: 5′-ATCAGGGACATTCAATAGCGTAGC
Occludin	forward: 5′-AATTCTTCACTTCTAACAAATGGACCTC,

	Reverse: 5′-CACATCACAATAATGAGCATAGACAGG
Ribosomal protein (RP2)	Forward: 5′-AAGCTGAGGATGCTCAAAGG,<br>Reverse: 5′-CCCATTAAACTCCAAGGCAA
HPRT1	forward: 5′-TGACACTGGCAAAACAATGCA,<br>Reverse: 5′-GGTCCTTTTCACCAGCAAGCT
